# Therapeutic potential of red bean (*phaseolus vulgaris*) peptides: anticancer, antihypertension, and antioxidant activities

**DOI:** 10.1038/s41598-025-22843-0

**Published:** 2025-11-06

**Authors:** Sittiruk Roytrakul, Janthima Jaresitthikunchai, Sawanya Charoenlappanit, Siriwan Thaisakun, Narumon Phaonakrop, Suthathip Kittisenachai, Kanyaratt Supaibulwatana

**Affiliations:** 1 National Center for Genetic Engineering and Biotechnology, National Science and Technology Development Agency, Pathumthani, 12120 Thailand; 2 Faculty of Science, Mahidol University, Rama VI Rd, Ratchathewi, Bangkok, 10400 Thailand

**Keywords:** Anticancer peptide, Antihypertensive peptide, Antioxidant peptide, Protein hydrolysate, Biotechnology, Plant sciences

## Abstract

Red beans (*Phaseolus vulgaris*) are gaining recognition for their potential in functional foods and nutraceuticals. This study isolated bioactive peptides under 3 kDa from red bean protein hydrolysate, generated by pepsin digestion and purified using sequential cation exchange, anion exchange, and reverse-phase chromatography. The resulting hydrolysate exhibited anticancer activity. Following chromatographic fractionation and LC-MS sequencing, eight candidate peptides were synthesized and comparatively assessed for bioactivity. Remarkably, peptide RB-8 (LIIPATSTKFL) demonstrated significant anticancer activity against colorectal (CaCO2) and cervical (SiHa) cancer cells through distinct mechanistic pathways. Furthermore, RB-7 (RGSKQRQKRQW) showed potent antihypertensive and DPPH radical scavenging activities, RB-6 (RRLRILL) displayed the strongest ABTS radical scavenging activity, and RB-1 (NLRKLKRL) exhibited the highest FRAP. These findings highlight the potential of red bean-derived peptides as natural agents for managing non-communicable diseases (NCDs) and their applicability in the food industry.

## Introduction

 Non-communicable diseases (NCDs), including cardiovascular diseases, cancer, chronic respiratory diseases, diabetes, neurodegenerative disorders, and hypertension, pose a significant and growing global health challenge, accounting for an estimated 41 million deaths annually, or 74% of all fatalities^1^. Among these, cancer remains a critical area of research due to its high mortality, complex and multifaceted etiology, and substantial impact on patient well-being. Despite advancements in targeted therapies and immunotherapy, effective treatment and mitigation of adverse effects for many cancer types remain significant hurdles. Similarly, hypertension, a major risk factor for cardiovascular and other NCDs, contributes substantially to the global disease burden, often requiring comprehensive management strategies.

Red beans (*Phaseolus vulgaris*), a globally significant and widely consumed legume, have garnered increasing attention for their inherent functional components^2^. These beans are a rich source of bioactive compounds, including proteins, lipids, polysaccharides, and various phytochemicals, demonstrating potential health benefits in the context of diabetes, diet-related disorders (overweight, obesity), cardiovascular diseases, and cancer^3,4^. Their protein content may also contribute to satiety and weight management^5^. While nutritionally advantageous, red beans contain antinutritional factors, such as trypsin and chymotrypsin inhibitors, which can have negative implications, particularly with increased protein consumption^2,6^. Germination is one of the methods to minimize these antinutrients. In addition, germination offers a cost-effective method to mitigate these antinutrients and enhance the nutritional profile of legumes by improving protein digestibility and free amino acid content^7,8^. Furthermore, germination of red beans has been suggested as a potent strategy to elevate total antioxidant activity^9^.

Therapeutic peptides, including anticancer peptides (ACPs) and host defense peptides (HDPs), have emerged as promising and potentially safer drug agents against cancer due to their high selectivity and specificity^10^. Recent research indicates that ACPs can selectively target cancer cells while minimizing harm to normal physiological functions^11,12^ positioning them as a compelling therapeutic avenue. The significant interest in this area is underscored by the numerous peptide-based therapies targeting diverse tumors currently under preclinical and clinical evaluation^13,14^ highlighting the importance of discovering novel ACPs for cancer treatment.

This study aimed to investigate the anticancer properties of protein hydrolysates derived from red beans. Bioactive peptides with molecular weights under 3 kDa were purified using chromatographic techniques and their amino acid sequences determined by LC-MS. Subsequently, eight peptides were chemically synthesized and evaluated for their potential anticancer, antihypertensive, and antioxidant effects. The findings of this research may identify prototype molecules or foundational compounds for the development of therapeutic agents or functional food applications targeting NCDs.

## Results

### Anticancer activities of < 3 kDa peptides derived from pepsin hydrolysate

The MTT assay was employed to evaluate the anticancer activity of a < 3 kDa peptide-containing protein hydrolysate (100 µg/mL) obtained from germinated red beans. The results revealed that the peptic hydrolysate displayed significant anticancer efficacy, with inhibition rates ranging from 30.45% to 75.77% against a panel of human cancer cell lines (Table 1).

**Table 1:** Anticancer activity of germinated red bean hydrolysate containing peptides < 3 kDa after 24-hour treatment at 100 µg/ml. Results are expressed as inhibitory percentage (mean ± SD). All experiments were repeated three times to ensure the reliability of the data. Different small superscript letters within the column statistical show highly significant differences at *p* *≤* 0.01 analyzed by one-way ANOVA with Duncan Multiple Range Test.

Anticancer activity of peptic protein hydrolysate	Inhibitory percentage (means ± SD)	
CaCO2	59.35±0.01	d
HepG2	53.57±0.03	e
MCF-7	75.77±0.00	a
NCI-H460	32.07±0.00	f
SiHa	70.77±0.00	c
SW620	30.45±0.00	g
Vero cell	70.81±0.01	b

## Purification of bioactive peptides from protein hydrolysate

Identified anticancer peptides (ACPs) are typically short amino acid sequences often featuring two or more positively charged residues, notably arginine and lysine. The anticancer activity of a peptide is further influenced by its constituent amino acid residues, which contribute to cationic, hydrophobic, and amphiphilic properties crucial for forming helical structures^15–17^. To enrich for cationic peptides, the germinated red bean hydrolysate was initially subjected to cation exchange chromatography (CEx). The bound fraction from the CEx column was eluted with 1 M NaCl and subsequently passed through an anion exchange chromatography (AEx) column to remove anionic peptides. The unbound fraction from the AEx was then collected and further purified using stepwise elution on a hydrophobic C18 column. As detailed in Table 2, the peptide fraction eluted from the C18 column with 10% acetonitrile (ACN) exhibited the most pronounced anticancer activity. Consequently, this fraction was selected for amino acid sequence determination using LC-MS. Peptide sequences exhibiting high Andromeda scores and matching *Phaseolus vulgaris* peptides were subsequently synthesized via solid-phase peptide synthesis.

**Table 2:** Anticancer activity of ten purified fractions from germinated red bean hydrolysate, separated by hydrophobic column separated by hydrophobic column chromatography, following a 24-hour treatment. Data are presented as inhibitory percentage (mean ± SD), all experiments were repeated three times to ensure the reliability of the data. The influencing factors (fraction and peptic protein hydrolysate) on anticancer activity show highly significant differences at *p* *≤* 0.01 derived using one-way ANOVA with Duncan Multiple Range Test.

**Fraction**	**CaCO2**	**HepG2**	**MCF-7**	**NCI-H460**	**SiHa**	**SW620**	**Vero**	Factor A: Acetonitrile (%) fraction
10% Acetonitrile	62.34±0.02	51.30±0.00	69.38±0.00	60.57±0.01	56.05±0.00	60.15±0.00	56.26±0.01	**
20% Acetonitrile	20.45±0.00	15.58±0.01	39.79±0.02	37.53±0.00	23.93±0.02	36.14±0.00	24.35±0.00	**
30% Acetonitrile	29.43±0.00	21.75±0.00	36.80±0.00	32.07±0.00	−7.23±0.00	30.45±0.00	−6.60±0.00	**
40% Acetonitrile	1.75±0.03	17.86±0.00	7.84±0.00	6.89±0.00	−13.09±0.00	4.21±0.01	−12.42±0.02	**
50% Acetonitrile	−3.49±0.00	6.49±0.02	−4.12±0.01	8.08±0.01	−15.53±0.01	5.45±0.00	−14.84±0.00	**
60% Acetonitrile	22.19±0.00	16.88±0.00	6.70±0.00	46.79±0.00	−23.24±0.00	45.79±0.00	−22.50±0.00	**
70% Acetonitrile	20.45±0.00	22.40±0.00	−5.88±0.00	22.80±0.00	−3.91±0.00	20.79±0.00	−3.30±0.00	**
80% Acetonitrile	10.22±0.00	24.03±0.01	−0.93±0.03	18.53±0.00	21.00±0.02	16.34±0.00	21.44±0.03	**
90% Acetonitrile	2.49±0.00	−2.60±0.00	−11.03±0.00	8.79±0.00	12.99±0.00	6.19±0.01	13.48±0.00	**
100% Acetonitrile	24.19±0.00	36.04±0.00	7.73±0.00	25.89±0.02	−0.10±0.00	24.01±0.00	0.48±0.00	**
Factor B: Peptic protein hydrolysate	**	**	**	**	**	**	**	A x B **

**Table 3:** Amino acid sequence and predicted physicochemical properties of the synthetic peptide. Predicted secondary structure elements: H (alpha helix), C (random coil), E (beta sheet/extended strand).

Peptide name	Peptide sequence	Molecular weight (Da)	pI	Net charge	Hydrophobicity (%)	Helix (%)	Sheet (%)	Coil (%)	Secondary structure
**RB-1**	NLRKLKRL	1,039.6979	12.16	+ 4	23.04	37.50	0	62.50	CCCHHHCC
**RB-2**	DHPTGGL	695.3239	4.87	−0.9	11.51	0	0	100.00	CCCCCCC
**RB-3**	SPTPPKNVPF	1,082.5761	9.86	+ 1	20.37	0	0	100.00	CCCCCCCCCC
**RB-4**	GSEIRSTSDDVL	1,278.3493	3.54	−2	21.01	0	25	75.00	CCCEECECCCCC
**RB-5**	APANGSAF	733.7843	3.77	0	10.26	0	0	100.00	CCCCCCCC
**RB-6**	RRLRILL	939.2263	12.40	+ 3	29.83	28.57	0	71.43	CCCHHCC
**RB-7**	RGSKQRQKRQW	1,457.6743	12.42	+ 5	4.84	9.09	18.18	72.73	CCCECEHCCCC
**RB-8**	LIIPATSTKFL	1,203.5003	10.12	+ 1	34.16	18.18	0	81.82	CCCCCCHHCCC

**Table 4:** Anticancer activity of synthetic peptides derived from purified protein hydrolysate of germinated red bean following 24-hour treatment at 100 µg/ml. Data are presented as inhibitory percentage (mean ± SD), all experiments were repeated three times to ensure the reliability of the data. Different small superscript letters within same column statistical show highly significant differences at *p* *≤* 0.01 analyzed by one-way ANOVA with Duncan Multiple Range Test.

Peptide name	Peptide sequence	CaCO2		HepG2	MCF-7		NCI-H460		SiHa		SW620		Vero
**RB-1**	NLRKLKRL	7.33±0.02	f	0	2.10±0.00	b	5.40±0.01	d	4.64±0.02	g	0	d	0
**RB-2**	DHPTGGL	8.99±0.08	e	0	0	c	0	f	10.28±0.02	d	0	d	0
**RB-3**	SPTPPKNVPF	7.88±0.08	f	0	0	c	0	f	12.36±0.02	b	0	d	0
**RB-4**	GSEIRSTSDDVL	11.27±0.07	d	0	0	c	6.08±0.01	c	11.45±0.00	c	0	d	0
**RB-5**	APANGSAF	13.21±0.04	c	0	0	c	10.58±0.03	a	8.75±0.00	f	3.58±0.04	b	0
**RB-6**	RRLRILL	19.32±0.18	b	0	0	c	0	f	9.72±0.02	e	0	d	0
**RB-7**	RGSKQRQKRQW	5.73±0.02	g	0	0	c	1.74±0.01	e	8.78±0.08	f	1.47±0.01	c	0
**RB-8**	LIIPATSTKFL	20.79±1.23	a	16.88±0.19	8.71±0.02	a	7.07±0.03	b	23.05±0.13	a	6.71±0.03	a	0

**Table 5:** The antihypertensive activity, expressed as the percentage Inhibition of the angiotensin-converting enzyme (ACE), and the antioxidant activity, expressed in µg ascorbic acid equivalents, were measured for the synthetic peptides (RB-1 to RB-8) at a concentration of 100 µg/ml. These peptides were derived from germinated red bean seeds. Data are presented as inhibitory percentage (mean±SD), all experiments were repeated three times to ensure the reliability of the data. Different small superscript letters within same column statistical show highly significant differences at *p* *≤* 0.01 analyzed by one-way ANOVA with Duncan Multiple Range Test.

Peptide name	Peptide sequence	Antihypertension (%)		Antioxidant (µg ascorbic acid equivalent)						
				ABTS			DPPH		FRAP	
**RB-1**	NLRKLKRL	20.00±2.82	d	367.86±25.39	c	373.74±12.01		d	189.53±6.59	a
**RB-2**	DHPTGGL	0	g	284.62±0.75	d	239.10±0.83		e	59.87±0.06	d
**RB-3**	SPTPPKNVPF	20.00±1.02	d	274.73±0.27	d	227.13±1.55		e	58.05±0.09	d
**RB-4**	GSEIRSTSDDVL	13.33±0.60	f	539.58±3.43	b	424.61±1.53		cd	128.47±0.86	b
**RB-5**	APANGSAF	43.33±0.33	c	412.60±0.71	c	374.62±13.56		d	87.48±0.20	c
**RB-6**	RRLRILL	46.67±0.36	b	595.33±18.41	a	584.66±4.15		b	120.46±0.07	b
**RB-7**	RGSKQRQKRQW	60.00±1.29	a	507.53±13.49	b	805.35±5.83		a	187.50±1.88	a
**RB-8**	LIIPATSTKFL	16.67±0.39	e	485.88±6.07	b	467.91±2.11		c	107.24±3.63	bc

## Physicochemical property of synthetic peptides derived from red bean hydrolysate

Following the purification of bioactive peptides with potent anticancer properties from germinated red bean protein hydrolysate using chromatographic methods, the fraction exhibiting the highest activity was subjected to liquid chromatography-tandem mass spectrometry (LC-MS/MS) for amino acid sequence determination. A total of eight peptides, characterized by high peptide scores and consisting of fewer than 12 amino acid residues, were selected for bioactivity evaluation (Table 2). These identified peptides were subsequently synthesized, and their physicochemical characteristics, including molecular weight (approximately 695.32–1457.67 Da), isoelectric point (pI 3.54–12.42), net charge (− 0.9 to + 5), and hydrophobicity (4.84–34.16%), are detailed in Table 2.

## Anticancer activity of synthetics peptides derived from pepsin hydrolysate

The peptic hydrolysate derived from germinated red beans exhibited varying degrees of anticancer efficacy across the human cancer cell lines (Table 3). The observed growth inhibition percentages were as follows: colon adenocarcinoma (CaCO2) cells (5.73–20.79%), hepatocellular carcinoma (HepG2) cells (0–16.88%), breast cancer (MCF-7) cells (0–8.71%), non-small cell lung cancer (NCI-H460) cells (0–10.58%), cervical carcinoma (SiHa) cells (4.64–23.05%), and colorectal cancer (SW620) cells (0–6.71%). Importantly, none of the synthesized peptides demonstrated any detectable toxicity towards monkey kidney Vero cells.

## Anticancer mechanism of RB-8 peptide

**Fig. 1:**
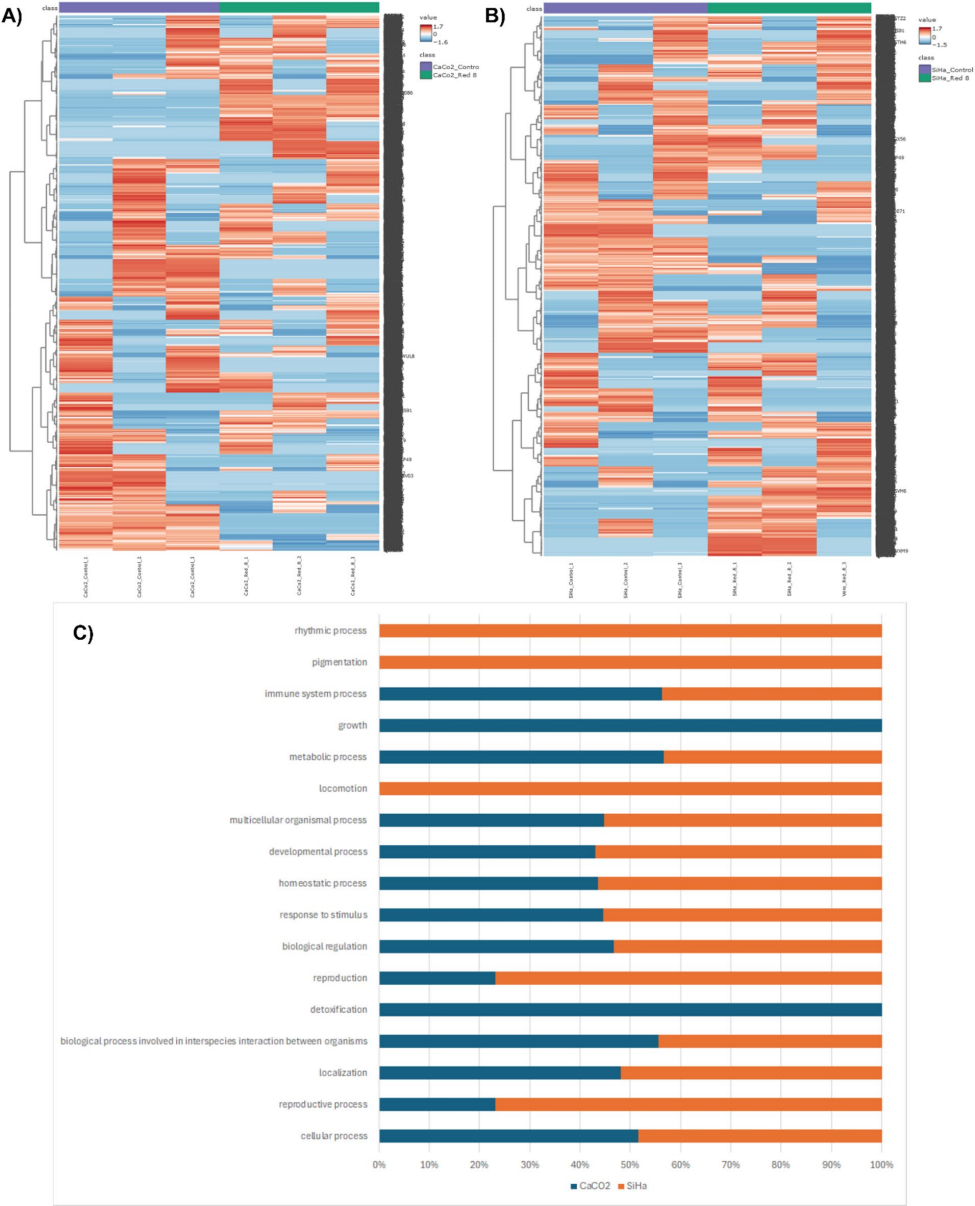
Heatmap visualization of the relative abundance of proteins identified in (**A**) Caco-2 and (**B**) SiHa cells after 24 h of exposure to 490.625 µg/ml RB-8 peptide. (**C**) Bar plot illustrating the enriched functional annotations of proteins that were significantly altered in abundance in Caco-2 and SiHa cells upon RB-8 peptide treatment.

The peptide RB-8 (LIIPATSTKFL) exhibited significant growth inhibitory activity against both colorectal (CaCO2) and cervical (SiHa) cancer cell lines, with a determined 50% inhibitory concentration (IC50) of 490.625 µg/ml for each. To elucidate the molecular mechanisms underlying these effects, a proteomic analysis was conducted on CaCO2 and SiHa cells following a 24-hour exposure to RB-8 at its IC50 concentration. Subsequent protein extraction, tryptic digestion, and mass spectrometry identified and quantified 12,312 proteins in CaCO2 cells and 13,196 proteins in SiHa cells (Fig. 1A and B). Functional annotation of these identified proteins using the PANTHER classification system, categorized by biological process (Fig. 1C), revealed distinct protein profiles in the two cell lines. In CaCO2 cells, the predominant protein functions were associated with growth and detoxification, whereas in SiHa cells, the major categories included rhythmic processes, pigmentation, and locomotion.

### Integrative approach for differential expression analysis

**Fig. 2:**
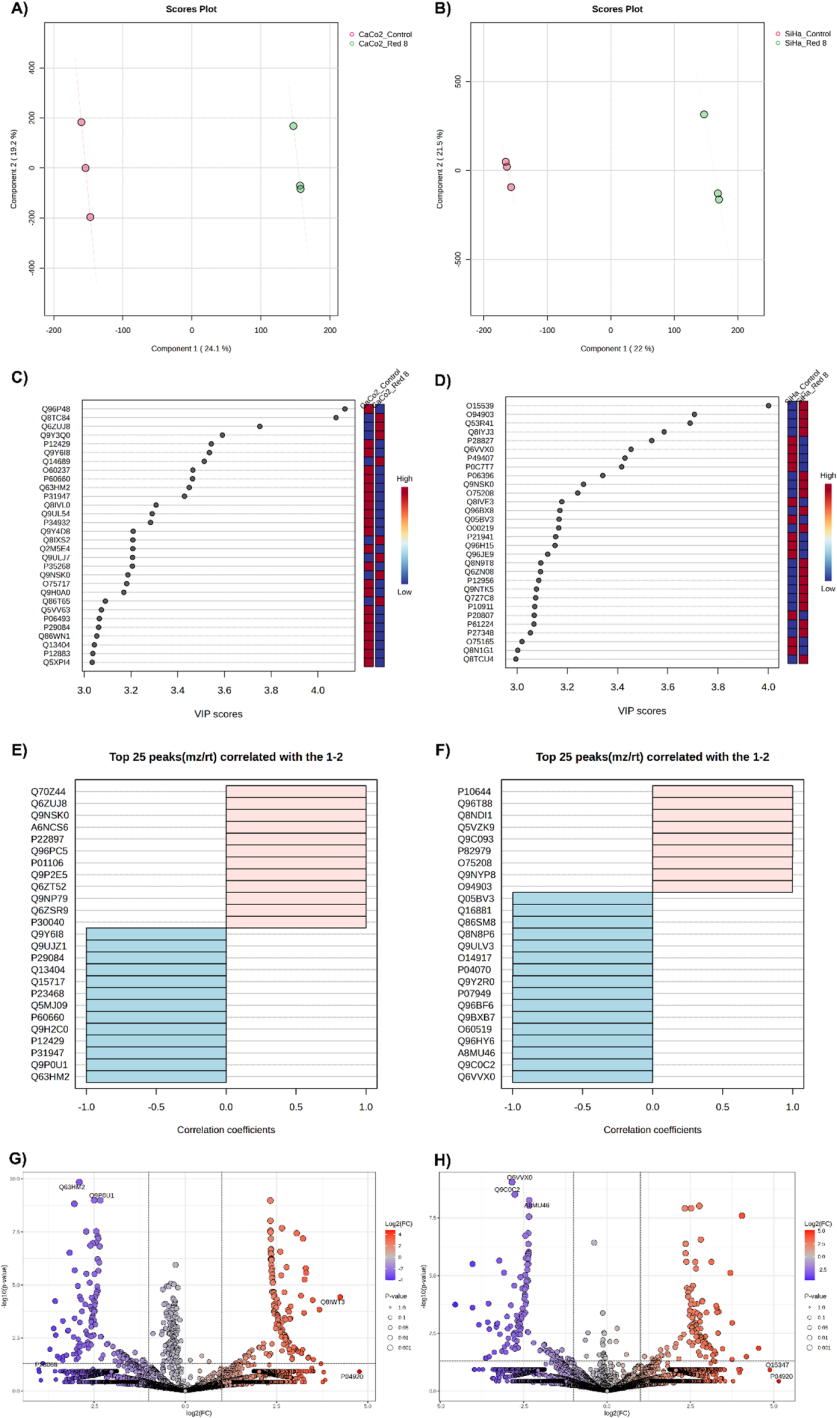
Statistical analysis of proteomic profiles in colon adenocarcinoma (CaCO2) and cervical squamous cell carcinoma (SiHa) cells following 24-hour exposure to 490.625 µg/ml of RB-8 peptide, relative to untreated control cells. (**A, B**) Two-dimensional partial least squares discriminant analysis (PLS-DA) score plots demonstrating the distinct clustering of untreated and RB-8 peptide-treated CaCO2 and SiHa cells. (**C, D**) PLS-DA variable importance in projection (VIP) score plots highlighting proteins contributing most significantly to the separation of experimental groups, ranked by their VIP score derived from the first principal component. Heatmaps depict the relative abundance of these discriminatory proteins within each group, with red and blue indicating high and low abundance, respectively. (**E, F**) Pearson correlation analysis of the top 25 differentially abundant proteins, illustrating positive (light pink) and negative (light blue) correlations in cells exposed to RB-8 peptide. (**G, H**) Volcano plots visualizing the significantly altered protein abundance in CaCO2 and SiHa cells after 24-hour exposure to the RB-8 peptide (*p* < 0.05, fold change > 1.5, unless otherwise specified in the main text).

Partial least squares discriminant analysis (PLS-DA) effectively distinguished the protein profiles of peptide-treated and untreated cells. To identify the key proteins driving the separation of CaCO2 and SiHa cells before and after RB-8 peptide exposure, multivariate PLS-DA was employed. The principal components derived from this analysis explained 43.3% and 43.5% of the variance for CaCO2 and SiHa cells, respectively (Fig. 2A and B). Variable Importance in Projection (VIP) scores, a critical metric from the PLS-DA models, were used to evaluate the contribution of individual proteins to the discrimination between experimental groups. The VIP-scored proteins and their corresponding relative expression levels in CaCO2 and SiHa cells before and after peptide treatment are depicted in Fig. 2C and D. Pearson correlation analysis was performed to assess the linear association between protein expression patterns in RB-8-treated CaCO2 and SiHa cells (Fig. 2E and F). Volcano plot analysis identified 676 and 644 proteins with significantly altered expression (p-value < 0.05 and fold change > 2) in CaCO2 and SiHa cells, respectively, following peptide exposure (Fig. 2G and H).

**Fig. 3:**
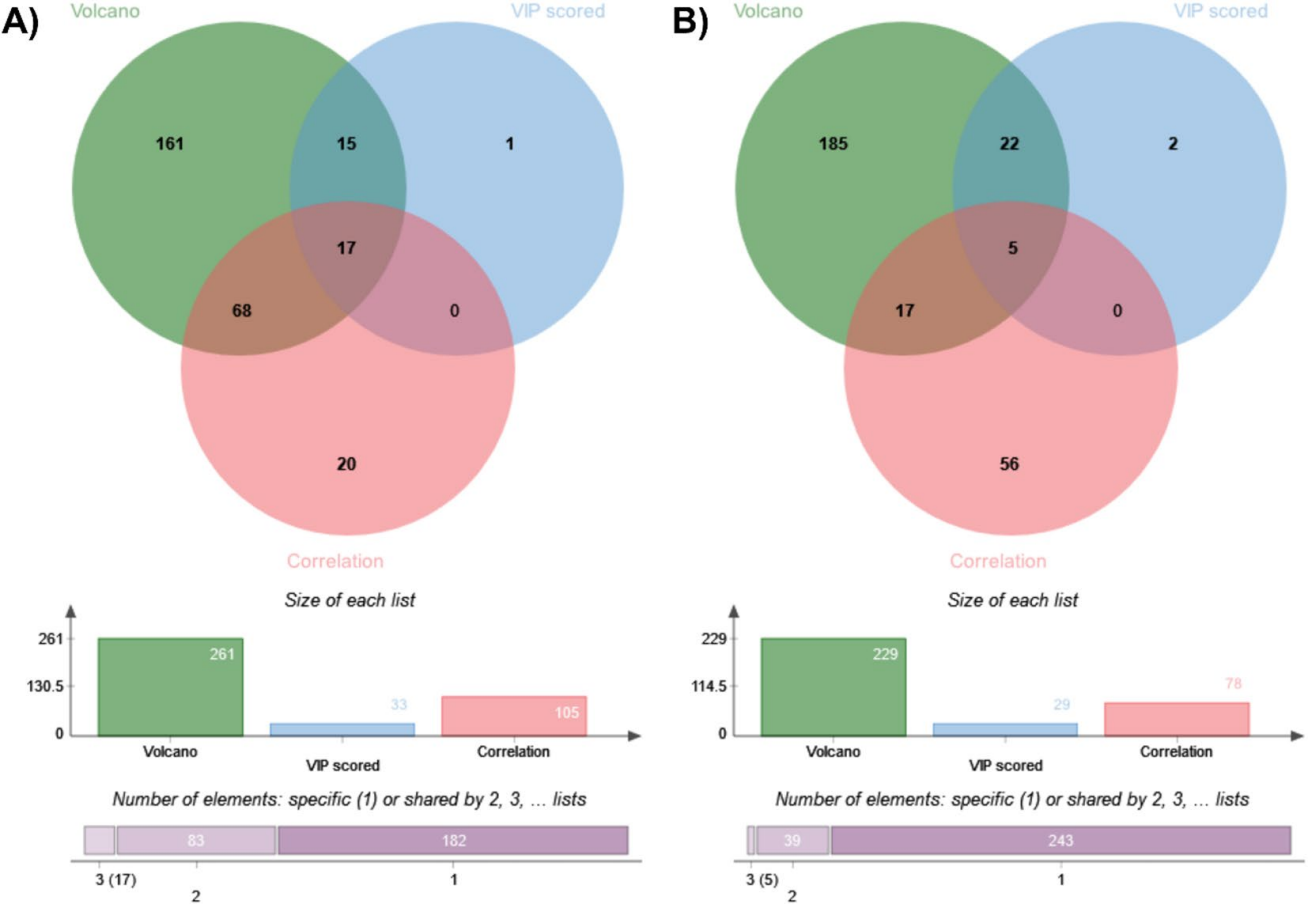
Venn diagrams displaying the intersection of significantly altered proteins in (**A**) CaCO2 and (**B**) SiHa cells treated with the RB-8 peptide. Protein differential expression was determined by a combined statistical approach employing volcano plots (*p* < 0.05; fold change > 2), partial least squares discriminant analysis (PLS-DA) variable importance in projection (VIP > 3.0), and Pearson correlation analysis (positive correlation: 0.99 ≤ *r* ≤ 1; negative correlation: −1 ≤ *r* ≤ − 0.99).

To refine the list of candidate proteins potentially mediating the effects of RB-8, the protein sets obtained from the Volcano plot analysis, PLS-DA VIP scores, and correlation analysis were integrated using a Venn diagram (Fig. 3). This integrated statistical approach applied the following stringent criteria: significant mean differences between groups (Volcano plot: p-value < 0.05 and fold change > 2), high discriminatory power (PLS-DA VIP scores > 3.0), and strong linear association (Pearson correlation coefficient [r]: 0.99 ≤ *r* ≤ 1 or −1 ≤ *r* ≤ −0.99). The integrative analysis revealed a distinct set of candidate proteins in each cell line following RB-8 treatment, specifically 17 proteins in CaCO2 cells and 5 proteins in SiHa cells (Fig. 3; Table 5).

**Table 6:** List of differentially expressed proteins identified in CaCO2 and SiHa cells following 24-hour exposure to 490.625 µg/ml RB-8 peptide, compared to untreated control cells.

CaCO2 cell						
**Uniprot/** **(Stitch) ID**	**Protein name**	**Function**	**Fold change (treated/control)**	**p-value**	**VIP score**	**Correlation**
Q9ULJ7 (ANKRD50)	Ankyrin repeat domain-containing protein 50	Endocytic recycling	9.6478	5.55E-06	3.2068	0.99808
Q9NSK0 (KLC4)	Kinesin light chain 4	Microtubule-based movement	6.2698	2.10E-08	3.1869	0.99988
Q6ZUJ8 (PIK3AP1)	Phosphoinositide 3-kinase adapter protein 1	Positive regulation of phosphatidylinositol 3-kinase/protein kinase B signal transduction	5.0364	9.43E-09	3.7514	0.99992
P31947 (SFN)	14-3-3 protein sigma	cAMP/PKA signal transduction	0.19937	1.02E-09	3.4289	-0.99997
P06493 (CDK1)	Cyclin-dependent kinase 1	Animal organ regeneration	0.1851	2.70E-05	3.0645	-0.99576
Q13404 (UBE2V1)	Ubiquitin-conjugating enzyme E2 variant 1	Cell differentiation	0.17254	1.09E-07	3.0431	-0.99973
P35268 (RPL22)	Large ribosomal subunit protein eL22	Alpha-beta T cell differentiation	0.16926	3.96E-05	3.2059	-0.99486
P29084 (GTF2E2)	Transcription initiation factor IIE subunit beta	Transcription by RNA polymerase II	0.16507	1.38E-07	3.0616	-0.9997
O75717 (WDHD1)	WD repeat and HMG-box DNA-binding protein 1	DNA repair	0.16299	7.80E-06	3.1817	-0.99772
Q8IVL0 (NAV3)	Neuron navigator 3	Negative regulation of cell migration	0.15296	2.97E-06	3.3074	-0.99859
P60660 (MYL6)	Myosin light polypeptide 6	Muscle contraction	0.15258	2.99E-08	3.4633	-0.99986
P34932 (HSPA4)	Heat shock 70 kDa protein 4	Chaperone-mediated protein complex assembly	0.13496	3.56E-05	3.2837	-0.99513
Q63HM2 (PCNXL4)	Pecanex-like protein 4	Positively regulates the expression of S-phase kinase associated protein 2	0.13381	1.43E-10	3.4494	-0.99999
Q5VV63 (ATRNL1)	Attractin-like protein 1	G protein-coupled receptor signaling pathway	0.12996	1.12E-05	3.0735	-0.99726
P12429 (ANXA3)	Annexin A3	Animal organ regeneration	0.12183	1.49E-09	3.5437	-0.99997
Q9Y6I8 (PXMP4)	Peroxisomal membrane protein 4	Ether lipid metabolic process	0.11137	3.02E-07	3.5357	-0.99955
Q96P48 (ARAP1)	Arf-GAP with Rho-GAP domain, ANK repeat and PH domain-containing protein 1	Actin filament organization	0.084631	5.80E-05	4.1158	0.99378
SiHa cell						
O94903 (PROSC)	Pyridoxal phosphate homeostasis protein	Influence brain function	8.7498	2.53E-06	3.7058	0.9987
Q9NTK5 (OLA1)	Obg-like ATPase 1	ATP metabolic process	7.2347	0.000133	3.0779	0.99056
P27348 (YWHAQ)	14-3-3 protein theta	Negative regulation of DNA-templated transcription	6.26	2.56E-06	3.0538	0.99869
P12956 (XRCC6)	X-ray repair cross-complementing protein 6	Activation of innate immune	5.5422	3.95E-05	3.0867	0.99487
O75208 (COQ9)	Ubiquinone biosynthesis protein COQ9, mitochondrial	Mitochondrial electron transport, NADH to ubiquinone	5.1159	1.07E-06	3.2418	0.99915

## Network analysis of CaCO2 and SiHa cells in response to RB-8 peptide

**Fig. 4:**
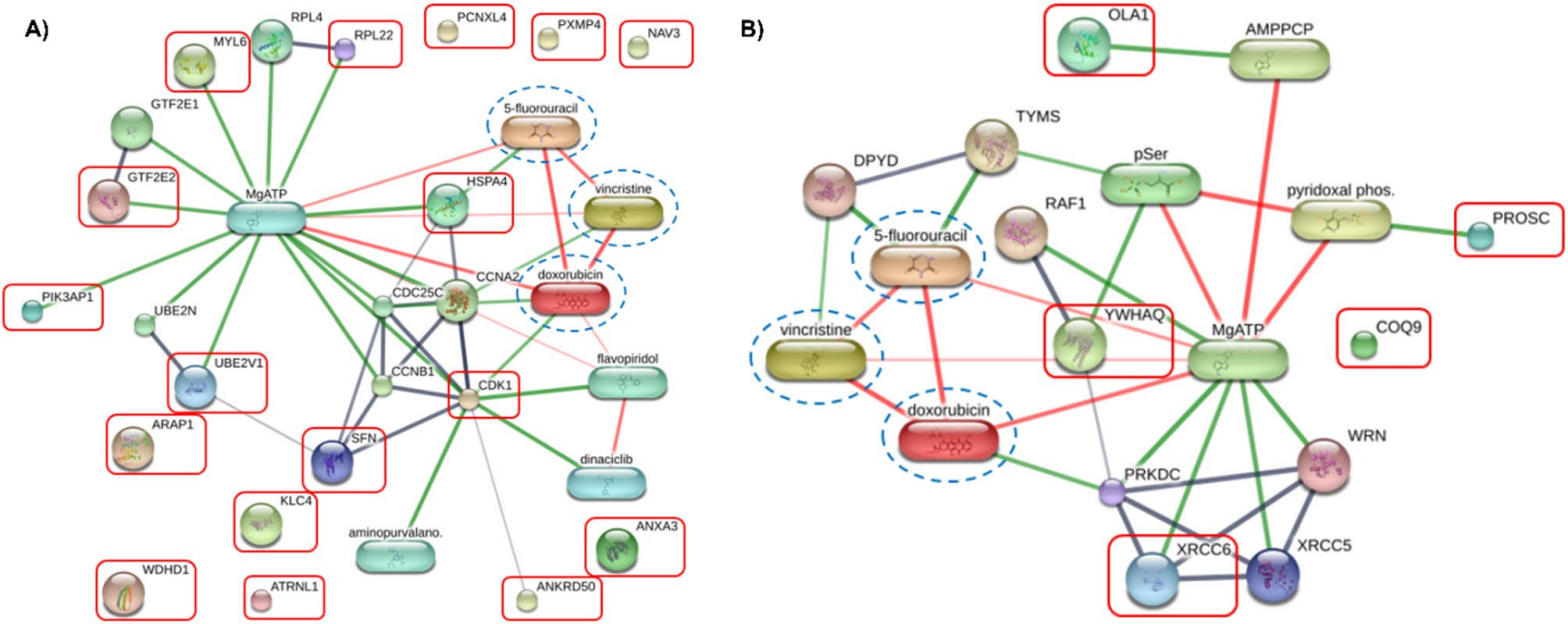
Protein-protein-chemotherapeutic drug interaction (PPCI) networks generated in (**A**) RB-8-treated CaCO2 cells and (**B**) RB-8-treated SiHa cells, illustrating potential interactions. Proteins identified in this study are marked with red squares. Chemotherapeutic drugs (5-FU, doxorubicin, and vincristine) are depicted as blue dashed circles.

Following the identification of candidate peptides, protein-chemical interactions were analyzed using the STITCH database to investigate potential mechanisms of action. Common chemotherapeutic drugs relevant to cancer treatment, specifically doxorubicin, vincristine, and 5-Fluorouracil (5-FU), were integrated into the analysis to predict associations and computational interactions. The resulting network analysis, illustrated in Fig. 4, demonstrated interactions between the identified proteins (9 out of 17 from RB-8-treated CaCO2 cells and 4 out of 5 from SiHa cells) and these chemotherapeutic agents. The protein-protein-chemotherapeutic drug interaction (PPCI) network revealed divergent responses to RB-8 peptide treatment in CaCO2 and SiHa cells, characterized by distinct patterns of protein-drug interactions. Interestingly, in CaCO2 cells (Fig. 4A), direct associations were identified between proteins such as HSPA4 and 5-FU, and CDK1 and doxorubicin, alongside indirect interactions involving RPL22, MYL6, GTF2E2, PIK3AP1, UBE2V1, SFN, and ANKRD50. In contrast, SiHa cells (Fig. 4B) exhibited only indirect interactions between the identified proteins XRCC6, YWHAQ, OLA1, and PROSC with the chemotherapeutic drugs. This differential PPCI landscape suggests that the RB-8 peptide likely exerts its effects on CaCO2 and SiHa cells through distinct mechanistic pathways, a finding that aligns with the enriched functional annotations of the significantly altered proteins in these cell lines (Fig. 1C).

## Antihypertensive and antioxidant activity of synthetic peptides derived from red bean hydrolysate

The synthetic peptides RB-1 to RB-8 demonstrated a range of antihypertensive activity, with angiotensin-converting enzyme (ACE) inhibition values spanning from 0% to 60%. The antioxidant properties of these peptides were evaluated using 2,2’-azino-bis(3-ethylbenzothiazoline-6-sulfonic acid) (ABTS), 2,2-diphenyl-1-picrylhydrazyl (DPPH), and ferric reducing antioxidant power (FRAP) assays. As detailed in Table 4, the ABTS radical scavenging activity of peptides RB-1 to RB-8 ranged from 274.73 to 595.33 µg ascorbic acid equivalent (AAE) g^−1^. The DPPH radical scavenging activity for the same peptides varied between 227.13 and 805.35 µg AAE g^−1^, while their FRAP values ranged from 58.05 to 189.53 µg AAE g^−1^. These results indicate that the antioxidant potential of the peptides was assay-dependent.

## Discussion

Red beans (*Phaseolus vulgaris*) are a globally significant dietary legume, recognized for their rich nutritional profile encompassing starches, dietary fiber, proteins, vitamins, and minerals, which contributes to their widespread availability^18,19^. The substantial protein content and favorable amino acid composition of this bean have attracted considerable interest from the food and nutraceutical industries as a promising source of hydrolysates and bioactive peptides^18–20^.

The germination process in red beans initiates a cascade of biochemical transformations, leading to elevated concentrations of key nutritional components, including protein, carbohydrates, peptides, amino acids, fatty acids, and glucose, compared to their non-germinated counterparts^21^. Moreover, the activation of diverse hydrolytic and proteolytic enzymes, such as amylases, proteases, and peptidases, during germination facilitates the breakdown of complex macromolecules into simpler forms that are more readily digestible and absorbable by the human body^22^. The potential of legume-derived peptides for various health applications is well-established. For example, peptides derived from soybean seeds have demonstrated efficacy in the management of diabetes and hypertension, exhibited anti-aging and weight loss properties, and have even been utilized as functional ingredients in cosmetics^23,24^. Corroborating these findings, research by Vermont et al. (2012)^25^ has confirmed the superior biological activity of protein hydrolysates obtained from germinated soybean seeds compared to those from non-germinated seeds.

To obtain bioactive peptides, the total protein isolated from germinated red bean seeds underwent enzymatic digestion with pepsin under acidic conditions, and the resulting peptide fraction with a molecular weight of less than 3 kDa was collected. The anticancer potential of this < 3 kDa protein hydrolysate derived from red bean seeds was subsequently evaluated. Particularly, these small peptides demonstrated inhibitory effects on the growth of a panel of human cancer cell lines, including colorectal (CaCO2), breast (MCF-7), hepatocellular carcinoma (HepG2), lung carcinoma (NCI-H460), cervical (SiHa), and colon (SW620) cells, as summarized in Table 1. This observation aligns with previous studies demonstrating the capacity of bean hydrolysates to inhibit the proliferation of various tumor cell lines^26–28^ with evidence suggesting that bean peptides within the 500–2000 Da range exhibit the most potent anticancer activity^29^.

Subsequent to the initial evaluation of anticancer activities, the < 3 kDa bioactive peptide fraction underwent a multi-stage purification process. This involved sequential fractionation using cation exchange chromatography, followed by anion exchange chromatography, and culminating in separation via a C18 column with stepwise acetonitrile gradient elution. The peptide fraction eluting at 10% acetonitrile demonstrated significant anticancer activities, as detailed in Table 2. Based on this promising bioactivity profile, this specific fraction was selected for subsequent peptide sequencing by LC-MS.

The anticancer bioactivity of peptides is well-established to be influenced by various physicochemical properties, including their molecular size, amino acid sequence, hydrophobicity, and net charge. The chemical and physical characteristics of eight synthetic peptides, designated RB-1 to RB-8, are summarized in Table 2. These synthetic peptides ranged in length from 7 to 12 amino acid residues and exhibited a range of net positive charges from − 2.0 to + 5.0. The anticancer activities of all designed peptides are detailed in Table 3, which aligns with previous reports highlighting the anticancer potential of low molecular weight peptides derived from beans^30,31^.

Among the synthesized peptides, RB-8 (LIIPATSTKFL), at a concentration of 100 µg/mL, exhibited superior anticancer activities compared to the other peptides (Table 3). This peptide displayed the highest hydrophobicity (34.16%) and a net charge of + 1, suggesting a potential correlation between increased hydrophobicity and enhanced anticancer efficacy. The presence of hydrophobic amino acid residues in short-chain peptides has been previously associated with anticancer potential^32,33^, as these residues can facilitate favorable interactions between anticancer peptides and the lipid bilayers of tumor cell membranes^29^. Conversely, the lower activity observed for the remaining synthetic peptides indicates that other factors, such as peptide chain length, net charge, and predicted helix content, did not necessarily correlate with improved anticancer activity within this series of peptides.

The peptide RB-8 (LIIPATSTKFL) demonstrated significant growth inhibitory activity against both colorectal (CaCO2) and cervical (SiHa) cancer cell lines, exhibiting a 50% inhibitory concentration (IC50) of 490.625 µg/ml for each. To elucidate the downstream effects of RB-8 at this concentration, a proteomic analysis was conducted on CaCO2 and SiHa cells following a 24-hour exposure. Subsequent protein extraction, tryptic digestion, and mass spectrometry identified and quantified 12,312 protein types in CaCO2 cells and 13,196 proteins in SiHa cells (Fig. 1A and B). Functional annotation and biological pathway analysis of these differentially expressed proteins were performed using the PANTHER classification system version 19.0. This analysis revealed that the majority of proteins in CaCO2 cells were associated with growth and detoxification processes, while those in SiHa cells were primarily categorized under rhythmic processes, pigmentation, and locomotion (Fig. 1C).

To further evaluate differential protein expression, Volcano plots (*p* < 0.05, fold change > 2) and Partial Least Squares Discriminant Analysis (PLS-DA) were implemented to discern protein profiles between cell states before and after treatment with the RB-8 peptide (Fig. 2). Proteins exhibiting Variable Importance in Projection (VIP) scores exceeding 3 were considered significant contributors to the PLS-DA model (Fig. 2C and D). Correlation analysis, utilizing the Pattern Hunter feature, was performed to identify protein expression changes during RB-8 peptide exposure, with proteins ranked by Pearson correlation coefficient (Fig. 2E and F). Venn diagrams were generated to visualize the overlap and distinctions among protein lists derived from these different differential analyses (Fig. 3). This integrative approach identified 17 candidate proteins from RB-8-treated CaCO2 cells and 5 candidate proteins from RB-8-treated SiHa cells. Subsequent network analysis demonstrated interactions between a subset of these identified proteins (9 from CaCO2 and 4 from SiHa cells) and common chemotherapeutic drugs relevant to cancer treatment, namely doxorubicin, vincristine, and 5-Fluorouracil (5-FU), as depicted in Fig. 4. This differential protein-chemotherapeutic drug interaction landscape suggests that the RB-8 peptide likely exerts its effects on CaCO2 and SiHa cells via distinct mechanistic pathways, a finding consistent with the enriched functional annotations of the significantly altered proteins in these cell lines (Fig. 1C).

Furthermore, all eight synthesized peptides exhibited a spectrum of bioactivities, demonstrating both antihypertensive and antioxidant properties, as detailed in Table 4. Notably, peptide RB-8 (LIIPATSTKFL) displayed a multifaceted profile, exhibiting not only anticancer activity but also significant antihypertensive and antioxidant effects, as evidenced by ABTS, DPPH, and FRAP assays. Among the synthesized peptides, RB-7 (RGSKQRQKRQW) demonstrated the most potent antihypertensive activity and the highest DPPH radical scavenging capacity. Furthermore, RB-6 (RRLRILL) exhibited the strongest ABTS radical scavenging activity, while RB-1 (NLRKLKRL) presented the highest ferric reducing antioxidant power (FRAP). The observation of multiple bioactivities within single peptides aligns with previous findings. For instance, BmKn2 has been reported to possess antibacterial, anticancer, and antibiofilm activities^34–38^. Similarly, the peptide R14, isolated from *Oryza minuta*, demonstrated both anticancer and potent regulatory activity against IL-1β production in MSU crystal-induced inflammation^39,40^. Moreover, Pug-4, a recently identified peptide from pomegranate, highlights the potential for peptides to possess significant anti-inflammatory and anti-biofilm effects, specifically against *Streptococcus mutans* adhesion^41,42^.

## Materials and methods

### Protein hydrolysate preparation

Red bean seeds (*Phaseolus vulgaris*) were sourced from Mitrdee Sahaidee Company Limited, Thailand. Prior to germination, the seeds were imbibed in an aqueous solution for 8 h at ambient temperature. Subsequently, the imbibed seeds were placed on moistened tissue paper within closed containers and shielded from light. On the third day of germination, the seeds were ground into a fine powder using liquid nitrogen with a mortar and pestle. The powder was suspended in a 200 mM sodium acetate buffer (pH 4.0) at a ratio of 1:8 and then autoclaved at 121 °C for 15 min. The crude protein was centrifuged at 10,000 x *g* for 10 min, and the supernatant was collected. The protein concentration of the supernatant was determined using the Lowry method^43^, with bovine serum albumin (BSA) as the standard. Pepsin (BIO Basic Canada Inc), with an activity of 3000–3500 NFU/mg, was then added at an enzyme-to-substrate ratio of 1:20. The mixture was subsequently incubated at 37 °C for 16 h. The enzymatic reaction was terminated by heating the mixture to 100 °C for 10 min. The resulting suspension was then filtered through a 3 kDa molecular weight cut-off (MWCO) semipermeable membrane (Vivaspin 20, GE Healthcare, Chicago, UK) to isolate peptides with molecular weights below 3 kDa. Peptide concentration was subsequently quantified using the Bradford method^44^, employing BSA as the standard.

### Anticancer activity assay

#### Cell culture

CaCO2, HepG2, MCF-7, NCI-H460, SiHa, SW620, and Vero cells were obtained from the American Type Culture Collection (ATCC, Manassas, VA, USA). Cells were cultured in Dulbecco’s Modified Eagle’s Medium (DMEM; PAA, Pasching, Austria), supplemented with 10% (v/v) fetal bovine serum (Gibco, South America), 10 mM HEPES (HyClone, Utah, USA), 2 mM L-glutamine, and 100 U/mL penicillin and 100 µg/mL streptomycin. Cultures were maintained at 37 °C in a humidified atmosphere containing 5% CO₂.

#### Cell viability assay

The anticancer activity of protein hydrolysates and synthetic peptides against CaCO2, HepG2, MCF-7, NCI-H460, SiHa, SW620, and Vero cells were evaluated using the MTT assay. Cells were seeded into 96-well plates (Nunc™, Roskilde, Denmark) at a density of 2 × 10⁴ cells per well, and allowed to adhere. Following seeding, cells were treated with 100 µg/mL of protein hydrolysates or synthetic peptides for 24 h, while untreated cells served as controls. After 24 h, 20 µL of MTT solution (5 mg/mL; Sigma, St. Louis, MO, USA) was added to each well and incubated for an additional 3 h. The supernatant was then removed, and 100 µL of DMSO was added to each well to dissolve the formazan crystals. Optical density (OD) was measured at 540 nm using a microplate reader (Rayto RT-2100 C, China). Cell viability was calculated using the equation: (OD of treated cells/OD of untreated cells) × 100.

The IC50 values were calculated from a fitted semi-log response curve, plotting cell viability percentages against concentration. These values were determined using the AAT-Bioquest IC50 calculator (https://www.aatbio.com/tools/ic50-calculator).

### Angiotensin I-converting-enzyme (ACE) inhibitory activity assay

The method utilized here is a spectrophotometric method based on the commonly used method introduced by Cushman and Cheung (1971)^45^. The 5 ul of sample was preincubated with 45 ul of ACE enzyme (0.2 U/mL) in 100 mM Phosphate buffer (pH 6.8) and incubated for 10 min at 37 °C. Enzyme activity was assayed by monitoring the hydrolysis of hippuryl-L-histidyl-L-leucine (HHL). After adding 50 µL of a 5 mM HHL substrate solution, the increase in absorbance at 405 nm was measured every minute for 15 min at 37 °C using a 96-well microplate reader. The initial velocity was determined from the linear portion of the curve, and the inhibitory activity was expressed as a percentage calculated using the following equation:

% inhibition = (1-(B-b)/(A-a)) x 100,

where *A* is an initial velocity of the control reaction with enzyme (control), *a* is an initial velocity of the control reaction without enzyme (control blank), *B* is an initial velocity of the enzyme reaction with sample and *b* is an initial velocity of the reaction with sample but without enzyme (sample blank).

### Antioxidant activity assay

#### ABTS assay

Radical scavenging antioxidant activity was determined according to the ABTS radical scavenging activity following the method described by Re et al.^46^ with slight modifications. Briefly, the solution of ABTS radical was prepared from the reaction between 7 mM ABTS (2,2-azino-bis (3-ethylbenzothiazoline-6-sulphonic acid) diammonium salt) and 2.45 mM ammonium persulphate. The resulting ABTS solution was diluted with distilled water to achieve an optical density of 0.7 at 750 nm. Then, a sample or standard of 10 µL was mixed with 190 µL of ABTS solution and incubated for 5 min in the dark. The absorbance was measured at 750 nm by a microplate reader (Rayto RT-2100 C, China). Ascorbic acid was used as the standard. Antioxidant capacity was exhibited as micrograms ascorbic acid equivalent (µg AAE·g^−1^).

#### FRAP assay

The ferric ion-reducing antioxidant power assay was analyzed according to the method by Rumpf et al.^47^ with minor modifications. Briefly, the FRAP reagent was prepared from 300 mM sodium acetate (pH 3.6) and 10 mM TPTZ (2,4,6-tris (2-pyridyl)-s-triazine) in 40 mM HCl and 20 mM ferric chloride at the ratio of 10:1:1, respectively. Then, a 10 µL aliquot of the sample or standard was mixed with 190 µL of the FRAP reagent before incubating in the dark for 30 min. The absorbance was measured at 593 nm using a microplate reader (Rayto RT-2100 C, China). Ascorbic acid was used as the standard. Results were expressed as micrograms of ascorbic acid equivalent per gram (µg AAE g^−1^).

### DPPH radical scavenging assay

The DPPH radical scavenging activity of the sample was evaluated following the protocol by Muangrod et al.^48^ with slight modifications. Briefly, 190 µL of 0.1 mM DPPH was mixed with 10 µL of the sample or standard ascorbic acid in each well and incubated at room temperature in the dark for 30 min. The absorbance of the mixture was measured at 517 nm using a microplate reader (Rayto RT-2100 C, China). Ascorbic acid was used as the standard. Results were expressed as micrograms ascorbic acid equivalent (µg AAE g^−1^).

### Purification of bioactive peptides from protein hydrolysates by chromatographic technique

The protein hydrolysate exhibiting anticancer properties underwent a sequential purification process using ion exchange chromatography (both cationic and anionic) followed by reverse-phase chromatography. Initially, the hydrolysate was passed through cation exchange chromatography (HiTrap SP FF Sepharose™) and then through anion exchange chromatography (HiTrap Q FF Sepharose™), with peptides eluted from both columns using 1 M sodium chloride (NaCl). The eluent from the cation exchange column was subsequently desalted and applied to the anion exchange column, yielding an unbound fraction which was further processed on a C18 reverse-phase column. Elution was performed in a stepwise gradient of acetonitrile concentrations (10%, 20%, 30%, 40%, 50%, 60%, 70%, 80%, 90%, and 100%). Each fraction was assayed for anticancer activity, with active fractions selectively collected for peptide sequencing via LC-MS.

### Peptide sequencing by LC-MS

The bioactive peptide fraction was purified using a C18 ZipTip (Merck Millipore) before concentration determination by the Bradford method with bovine serum albumin as the standard. Peptide samples were analyzed by LC-MS using a Thermo Scientific Ultimate3000 Nano/Capillary LC system coupled to a Bruker impact II™ Q-Tof mass spectrometer with a Nano-captive spray ion source. One microliter of each peptide sample was separated on a 75 μm i.d. × 15 cm Acclaim PepMap RSLC C18 column at 60 °C. Elution was performed over 30 min (5–55% solvent B) using a gradient of 0.1% formic acid (A) and 0.1% formic acid in 80% acetonitrile (B) at 0.3 µL/min. Electrospray ionization (1.6 kV, nitrogen drying gas) and collision-induced dissociation (nitrogen collision gas, 10 eV collision energy) were used to acquire positive-ion mode product mass spectra (m/z 150–2200) at 2 Hz. Each sample underwent LC-MS analysis in triplicate. Peptide sequence analysis was performed using the Andromeda search engine by matching MS/MS spectra against the *Phaseolus vulgaris* Uniprot database.

### Peptide synthesis

Peptide synthesis was carried out by GenScript (Piscataway, NJ, USA) using Fmoc solid-phase synthesis. All synthesized peptides exhibited a purity of over 95%, as confirmed by mass spectrometry analysis. Physiochemical properties, including molecular weight and hydrophobicity, were calculated using the Thermo Fisher Scientific Peptide Analyzing Tool (Thermo Fisher Scientific, Peptide Analyzing Tool, available at Thermo Fisher Scientific website), while isoelectric point and net charge were determined using the INNOVAGEN Peptide Property Calculator^49^. Secondary structure attributes, such as helix and coil percentages, were predicted using the GOR method (GOR IV) through the GORI online tool^50^.

### Shotgun proteomics analysis of cancer cells after exposure to RB-8 peptide

Cell lysis was performed using a minimal volume of 0.5% solution of Sodium dodecyl sulfate (SDS), followed by thorough vortexing and centrifugation at 10,000 g for 15 min. The resulting supernatant was carefully transferred to a fresh tube, thoroughly mixed with two volumes of ice-cold acetone, and incubated overnight at −20 °C to facilitate protein precipitation. Following precipitation, the mixture was centrifuged at 10,000 g for 15 min, and the supernatant was discarded. The protein pellet was then dried and stored at −80 °C until further processing. Protein concentration of the samples was determined using the Lowry assay, with bovine serum albumin (BSA) as the protein standard^43^.

For in-solution digestion, five micrograms of protein samples were completely solubilized in 10 mM ammonium bicarbonate (AMBIC). Disulfide bonds were reduced by incubation with 5 mM dithiothreitol (DTT) in 10 mM AMBIC at 60 °C for 1 h. Subsequently, sulfhydryl groups were alkylated by incubation with 15 mM iodoacetamide (IAA) in 10 mM AMBIC at room temperature for 45 min in the dark. The protein samples were then digested with sequencing-grade porcine trypsin (enzyme: protein ratio of 1:20) for 16 h at 37 °C. The resulting tryptic peptides were dried using a speed vacuum concentrator and resuspended in 0.1% formic acid prior to LC-MS analysis.

Tryptic peptide samples were prepared for injection into an Ultimate3000 Nano/Capillary LC System (Thermo Scientific, UK) coupled online to a Hybrid quadrupole Q-Tof impact II™ mass spectrometer (Bruker Daltonics) equipped with a Nano-captive spray ion source. Briefly, one microlitre of the peptide digests was loaded onto a µ-Precolumn (300 μm i.d. x 5 mm, C18 Pepmap 100, 5 μm, 100 Å; Thermo Scientific, UK) for enrichment and desalting. Subsequent separation was performed on a reversed-phase analytical column (75 μm I.D. x 15 cm) packed with Acclaim PepMap RSLC C18 (2 μm, 100 Å, nanoViper; Thermo Scientific, UK), maintained at 60 °C using a thermostatted column oven. Mobile phases consisted of 0.1% formic acid in water (Solvent A) and 0.1% formic acid in 80% acetonitrile (Solvent B), delivered at a constant flow rate of 0.30 µl/min. Peptide elution was achieved using a linear gradient of 5–55% Solvent B over 30 min. Electrospray ionization was conducted at 1.6 kV using the CaptiveSpray source, with nitrogen employed as the drying gas at a flow rate of approximately 50 l/h. Collision-induced dissociation (CID) product ion mass spectra were acquired using nitrogen as the collision gas. Mass spectra (MS) and MS/MS spectra were obtained in positive-ion mode at a data acquisition rate of 2 Hz across the m/z range of 150–2200. The collision energy was dynamically adjusted to 10 eV as a function of the precursor m/z value. For each sample, LC-MS analysis was performed in triplicate.

### Bioinformatics and data analysis

Protein quantification in individual samples was performed using MaxQuant software (version 2.6.6.0) and the integrated Andromeda search engine. MS/MS spectra was correlated to the UniProt *Homo sapiens* database^51^. Label-free quantification was conducted using MaxQuant’s default parameters, with the following key settings: a maximum of two missed tryptic cleavages allowed; a mass tolerance of 0.6 Da for the main search; carbamidomethylation of cysteine as a fixed modification; and oxidation of methionine and N-terminal acetylation as variable modifications. Protein identification required a minimum peptide length of 7 amino acids and at least one unique peptide. For subsequent data analysis, only proteins identified by at least two peptides, including at least one unique peptide, were considered. The protein false discovery rate (FDR) was set to 1% and estimated using a reversed database search strategy. A maximum of five modifications per peptide was permitted. The *Homo sapiens* proteome database downloaded from UniProt (April 17, 2025) served as the search FASTA file.

### Data analysis

Visualization and statistical analyses of the LC-MS data were conducted using MetaboAnalyst software^52^. This included the generation of heatmaps, partial least squares discriminant analysis (PLS-DA), and the assessment of Variable Importance in Projection (VIP) scores to identify the most discriminant proteins. Volcano plots were generated to identify proteins with significantly altered abundance, employing a cutoff p-value of < 0.05 and a fold change (FC) of > 2. Venn diagrams^53^ were utilized to illustrate the overlap and distinctions among protein lists derived from different differential analyses. Functional organization and biological role analysis of the differentially expressed proteins were performed using the PANTHER classification system (version 19.0)^54^. Furthermore, the STITCH database (version 5) was employed to analyze both known and predicted functional interaction networks between the identified proteins and chemotherapeutic agents^55^.

### Statistical analysis

All experiments were repeated three times to ensure the reliability of the data. The experimental data were expressed as mean ± standard deviation (SD). Microsoft Office Excel 365 was used for data analyses and graphs. Values of *p* < 0.01 were indicative of significant differences.

## Conclusion

Enzymatic hydrolysis of red bean proteins presents a compelling avenue for generating bioactive peptides with significant potential in functional foods and nutraceuticals. This study successfully isolated and purified a < 3 kDa peptide fraction from germinated red bean hydrolysate, demonstrating inherent anticancer activity. Subsequent identification and synthesis of eight candidate peptides revealed that RB-8 (LIIPATSTKFL) possesses a remarkable multi-faceted bioactivity profile, exhibiting anticancer effects against colorectal (CaCO2) and cervical (SiHa) cancer cells through distinct mechanisms, alongside antihypertensive and antioxidant properties. Furthermore, RB-1 (NLRKLKRL), RB-6 (RRLRILL), and RB-7 (RGSKQRQKRQW) demonstrated potent antihypertensive and distinct antioxidant activities, respectively. Collectively, these findings strongly underscore the potential of red bean-derived peptides as natural, multi-target agents for the management of non-communicable diseases and their promising integration into the food industry.

## Data Availability

The MS/MS raw data and analysis are available in the ProteomeXchange Consortium via the jPOST partner repository: JPST003793 and PXD063640. (https://proteomecentral.proteomexchange.org/cgi/GetDataset?ID=PXD063640)
